# Supplementary figures and images for: Stable flow-induced expression of KLK10 inhibits endothelial inflammation and atherosclerosis
Source: eLife. 2022 Jan 11;11:e72579. doi: 10.7554/eLife.72579 (PMC8806187; doi:10.7554/eLife.72579)

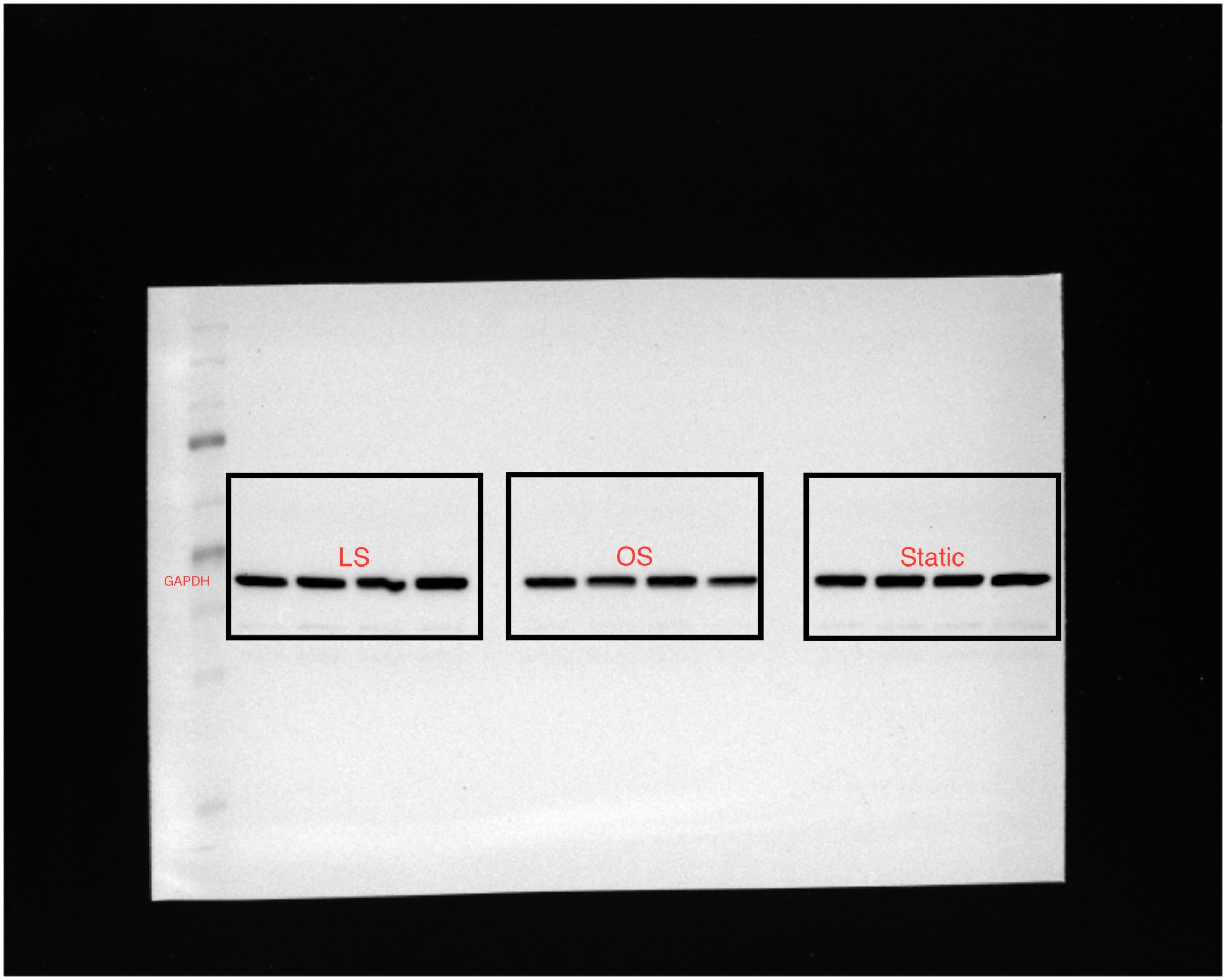

Supplement: Figure 1—source data 1. [file elife-72579-fig1-data1.zip › Figure 1- Source data 1/Figure1g-GAPDH_composite.tif]

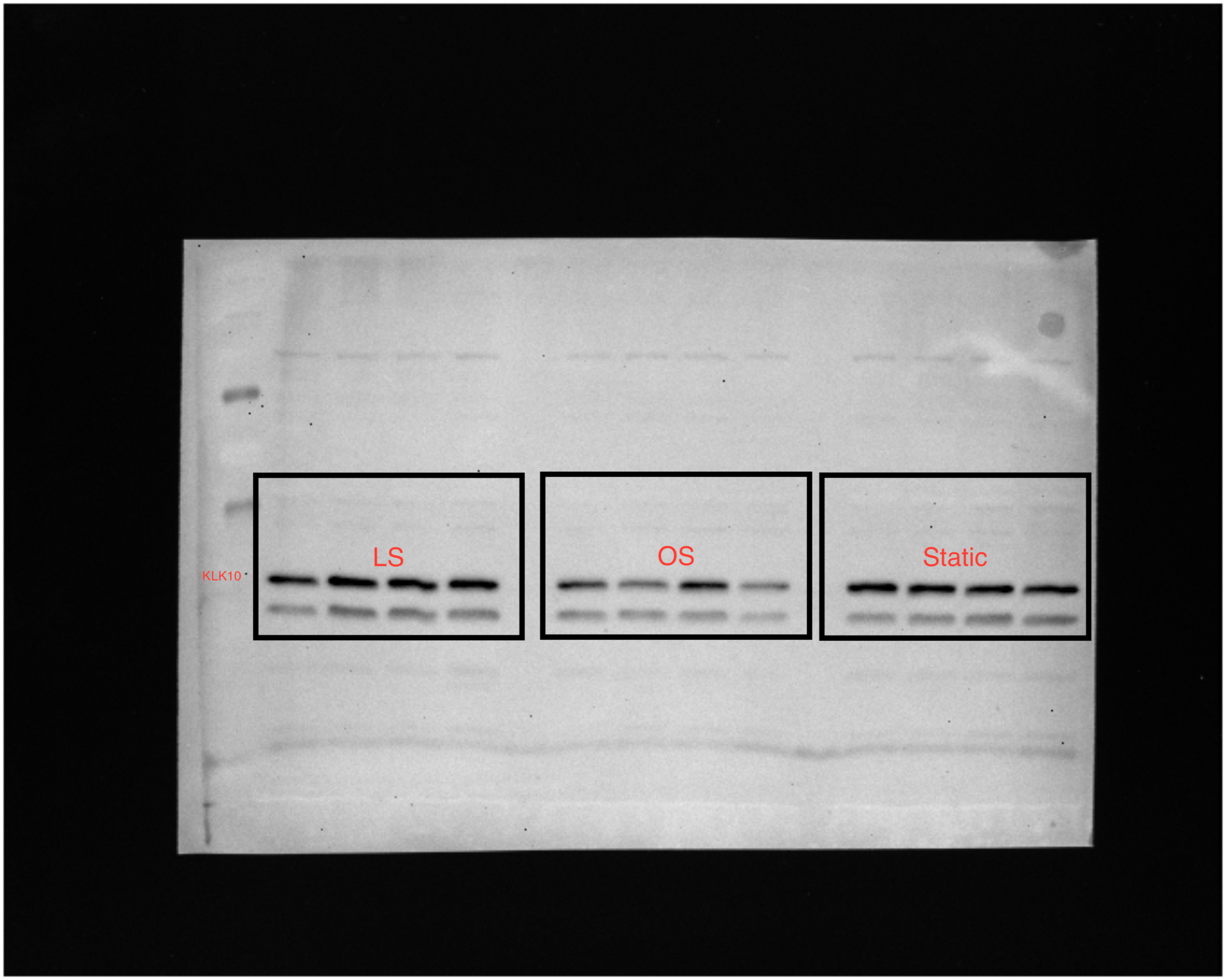

Supplement: Figure 1—source data 1. [file elife-72579-fig1-data1.zip › Figure 1- Source data 1/Figure1g-KLK10_composite.tif]

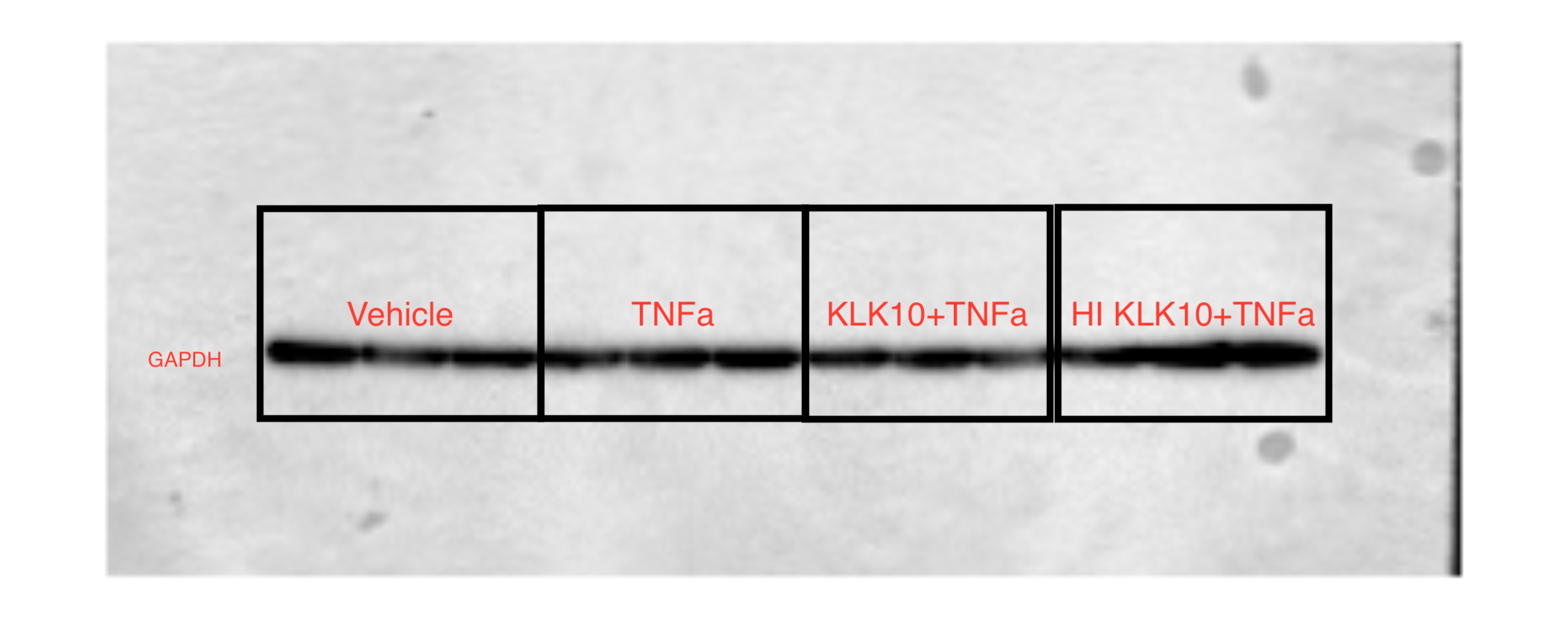

Supplement: Figure 2—source data 1. [file elife-72579-fig2-data1.zip › Figure 2- Source data 1/Figure2e-GAPDH_composite.tif]

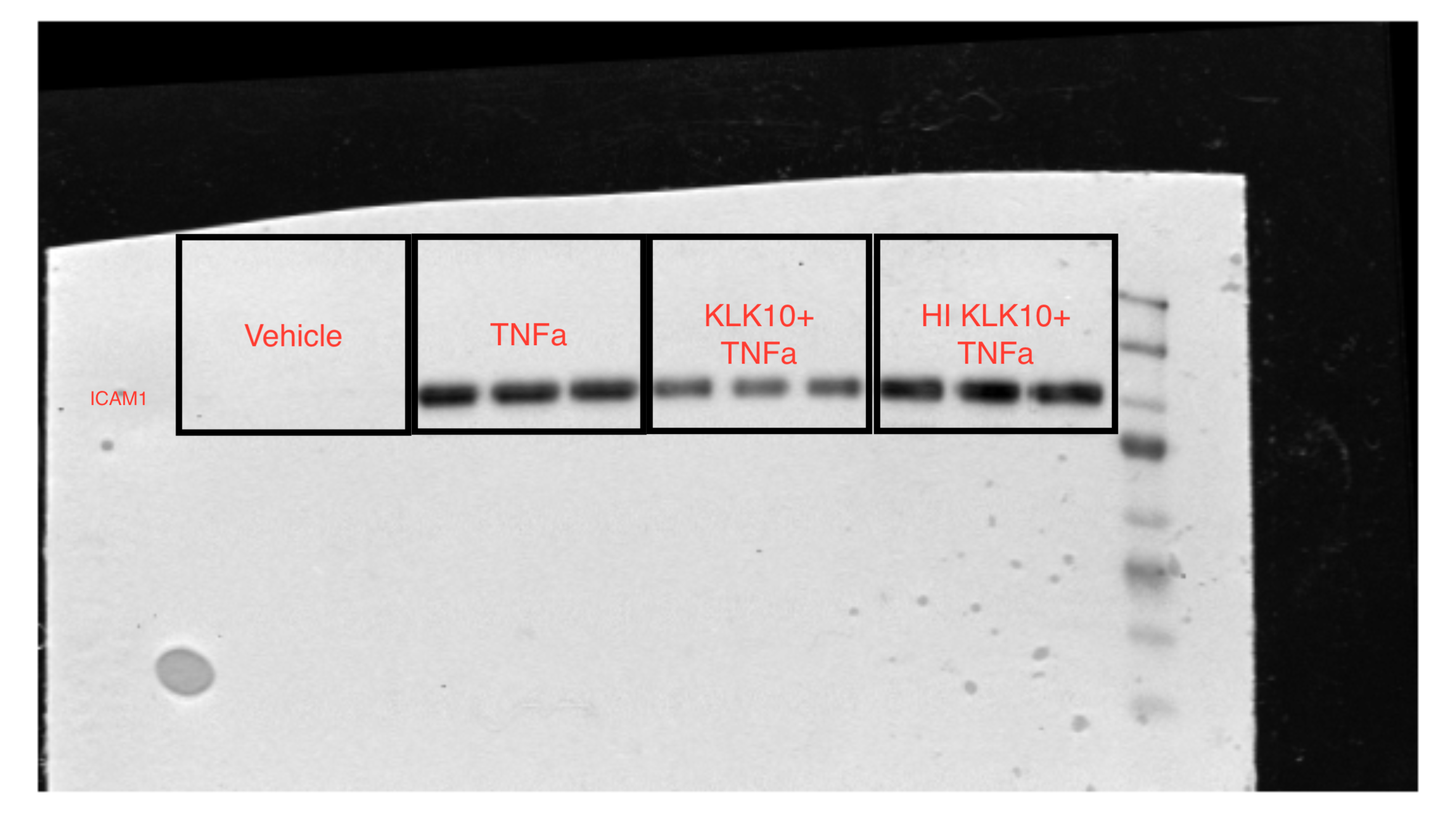

Supplement: Figure 2—source data 1. [file elife-72579-fig2-data1.zip › Figure 2- Source data 1/Figure2e-ICAM1_composite.tif]

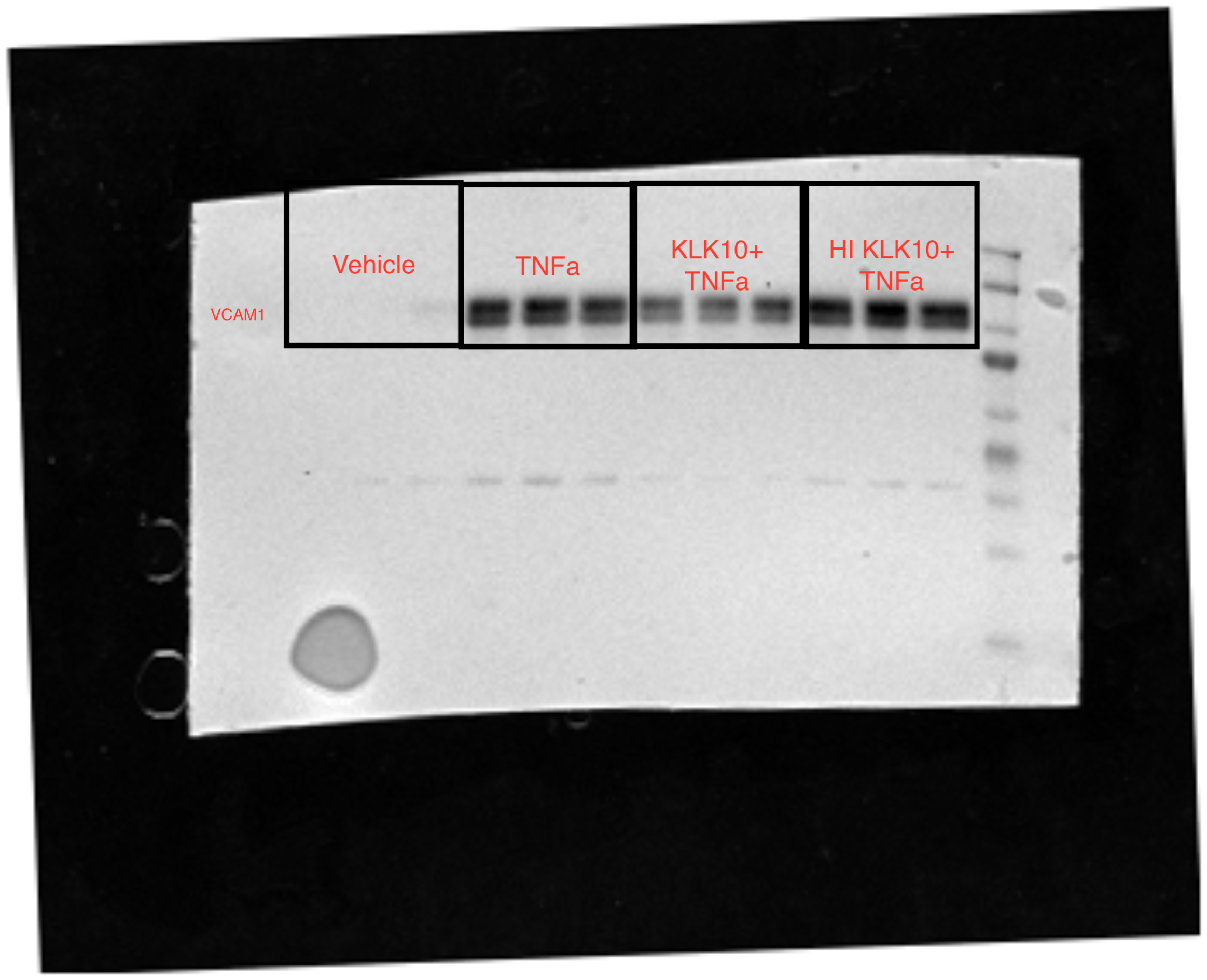

Supplement: Figure 2—source data 1. [file elife-72579-fig2-data1.zip › Figure 2- Source data 1/Figure2e-VCAM1_composite.tif]

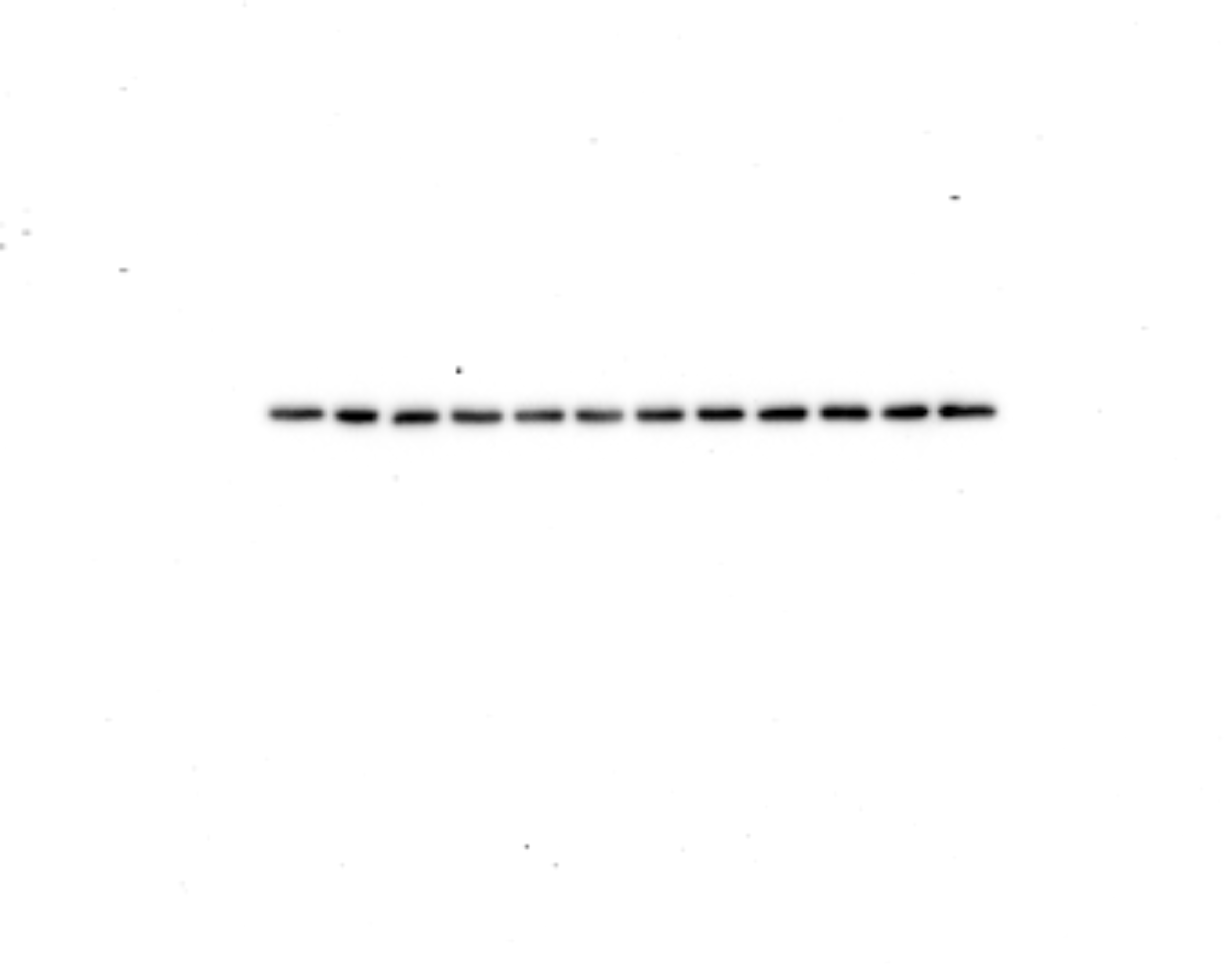

Supplement: Figure 2—figure supplement 6—source data 1. [file elife-72579-fig2-figsupp6-data1.zip › Figure 2- Figure Supplement 6- Source Data 1/GAPDH Western Blot.tif]

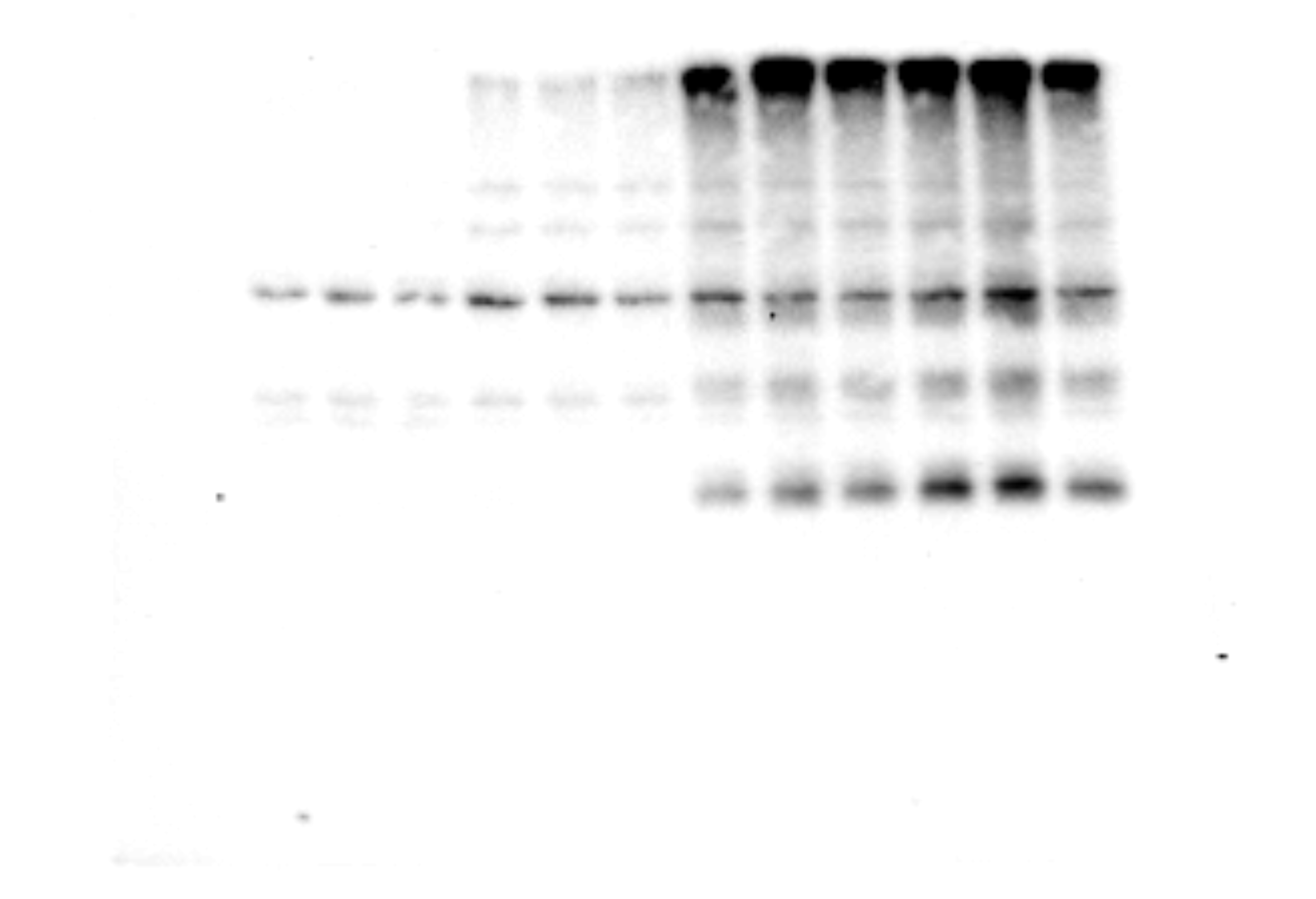

Supplement: Figure 2—figure supplement 6—source data 1. [file elife-72579-fig2-figsupp6-data1.zip › Figure 2- Figure Supplement 6- Source Data 1/His Western Blot.tif]

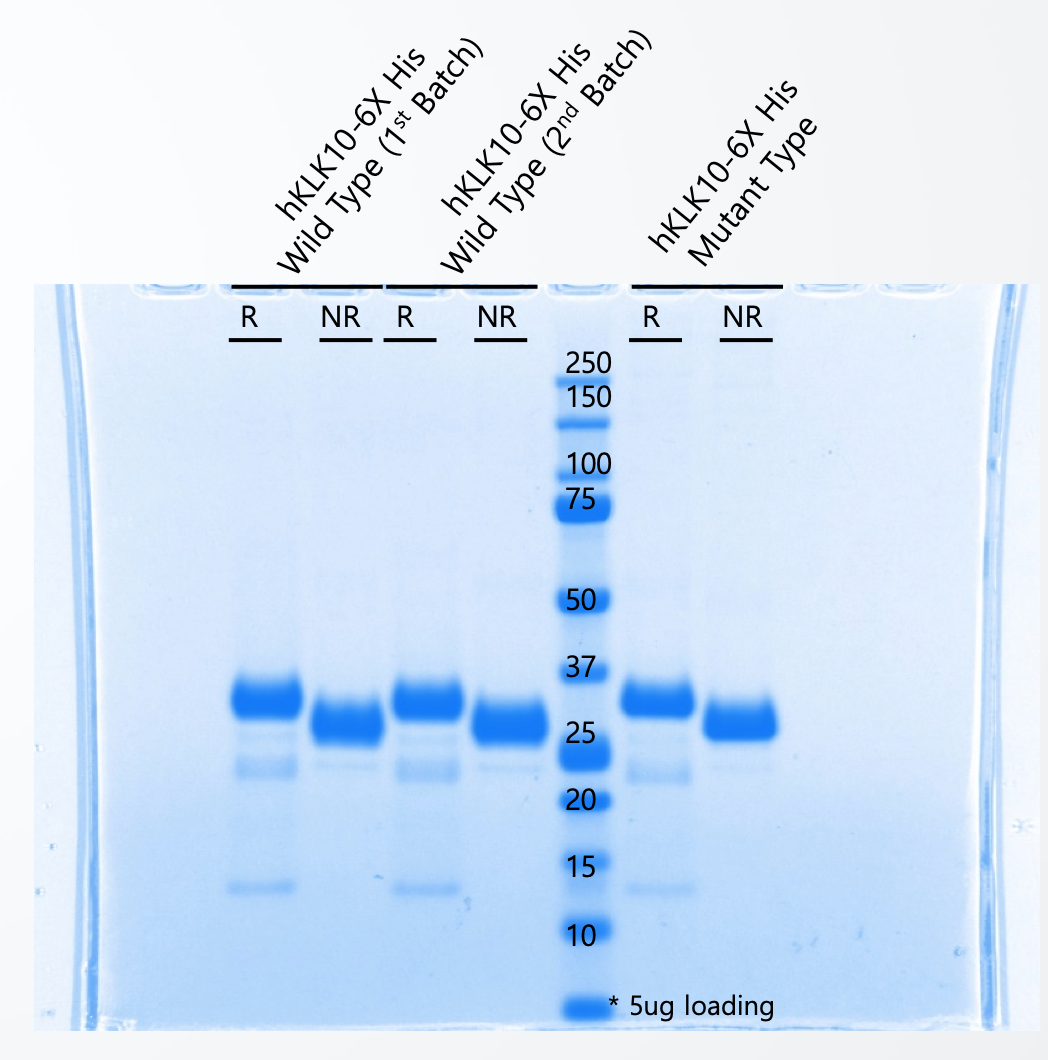

Supplement: Figure 2—figure supplement 7—source data 1. [file elife-72579-fig2-figsupp7-data1.zip › Figure 2- Figure Supplement 7- Source Data 1/Supplementary Figure 12.png]

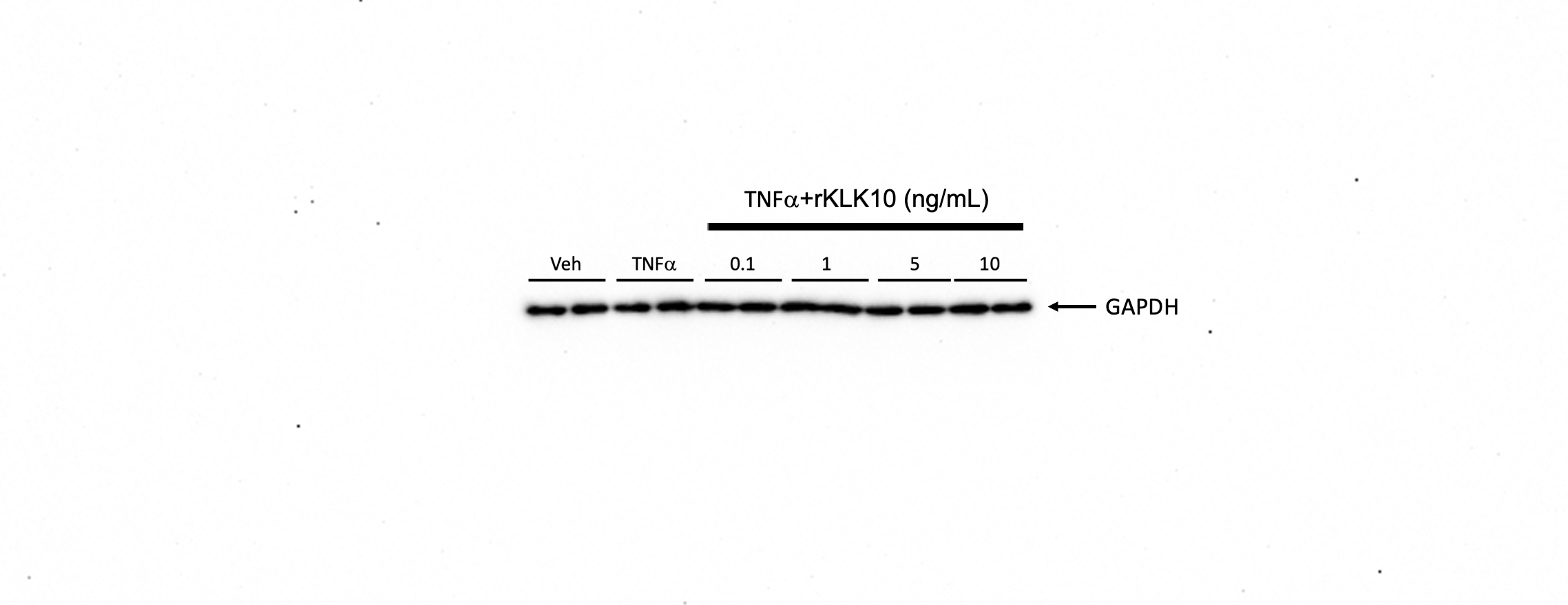

Supplement: Figure 3—source data 1. [file elife-72579-fig3-data1.zip › Figure 3- Source data 1/Figure 3a GAPDH n=1,2.tiff]

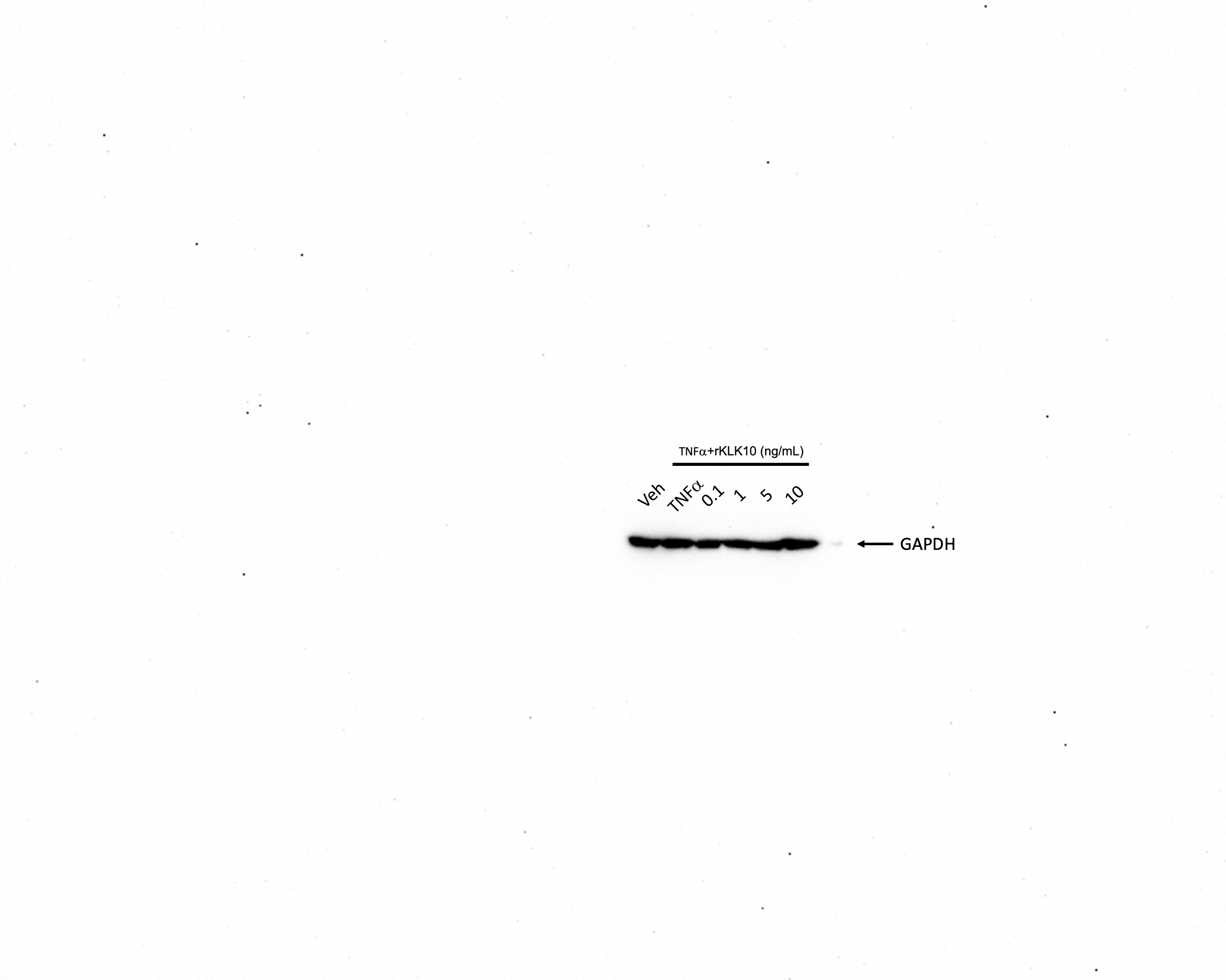

Supplement: Figure 3—source data 1. [file elife-72579-fig3-data1.zip › Figure 3- Source data 1/Figure 3a GAPDH n=3.tiff]

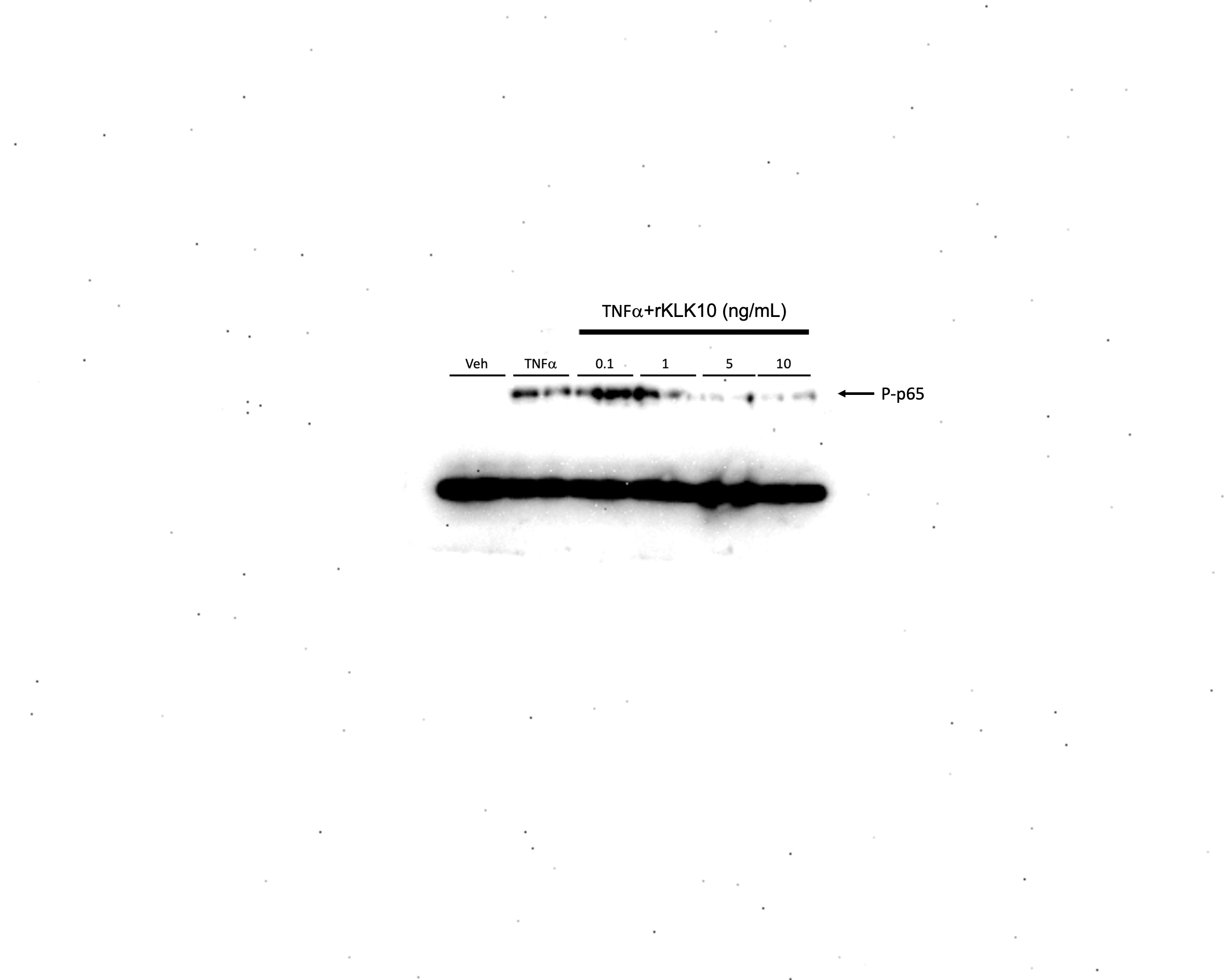

Supplement: Figure 3—source data 1. [file elife-72579-fig3-data1.zip › Figure 3- Source data 1/Figure 3a p-p65 n=1,2.tiff]

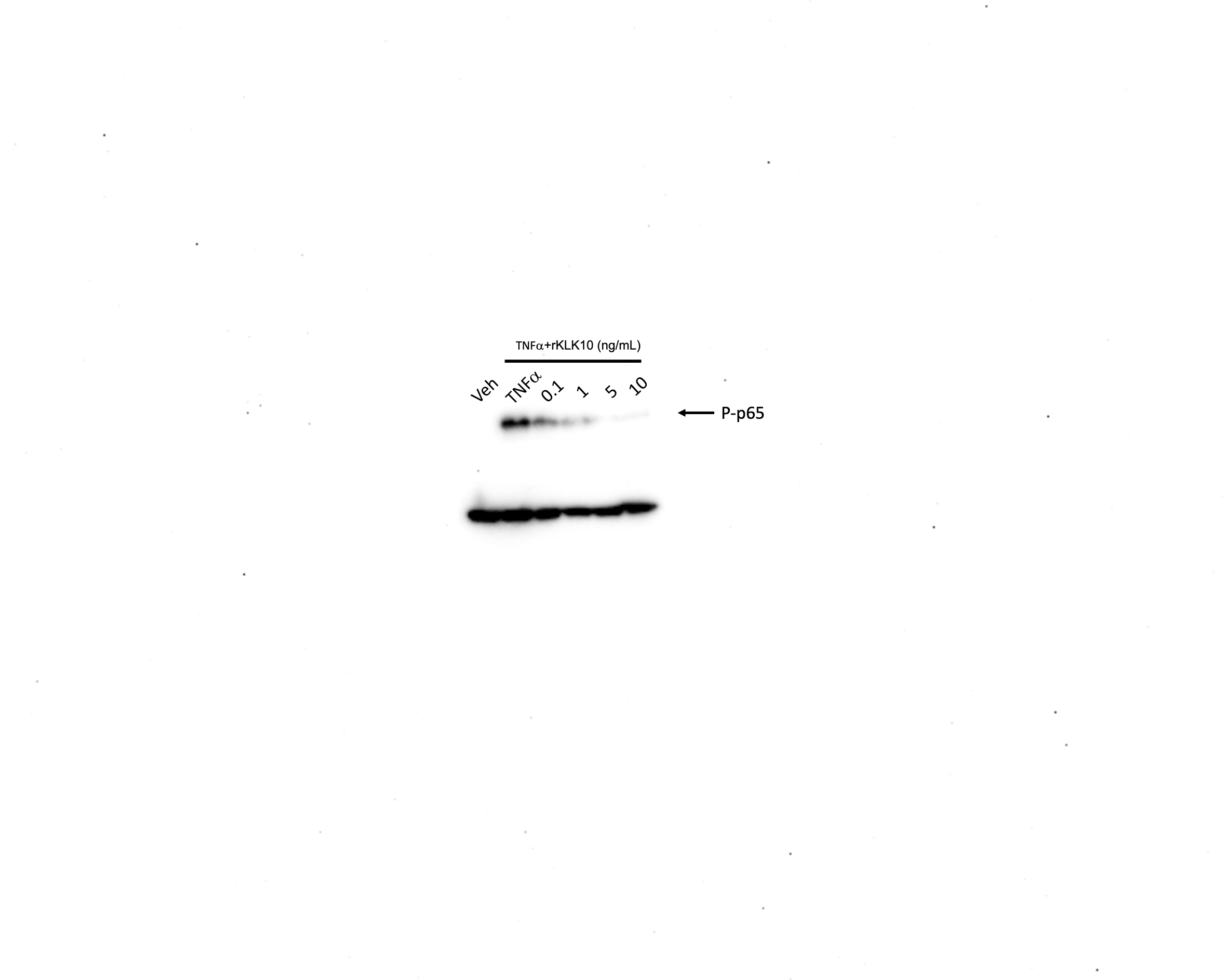

Supplement: Figure 3—source data 1. [file elife-72579-fig3-data1.zip › Figure 3- Source data 1/Figure 3a p-p65 n=3.tiff]

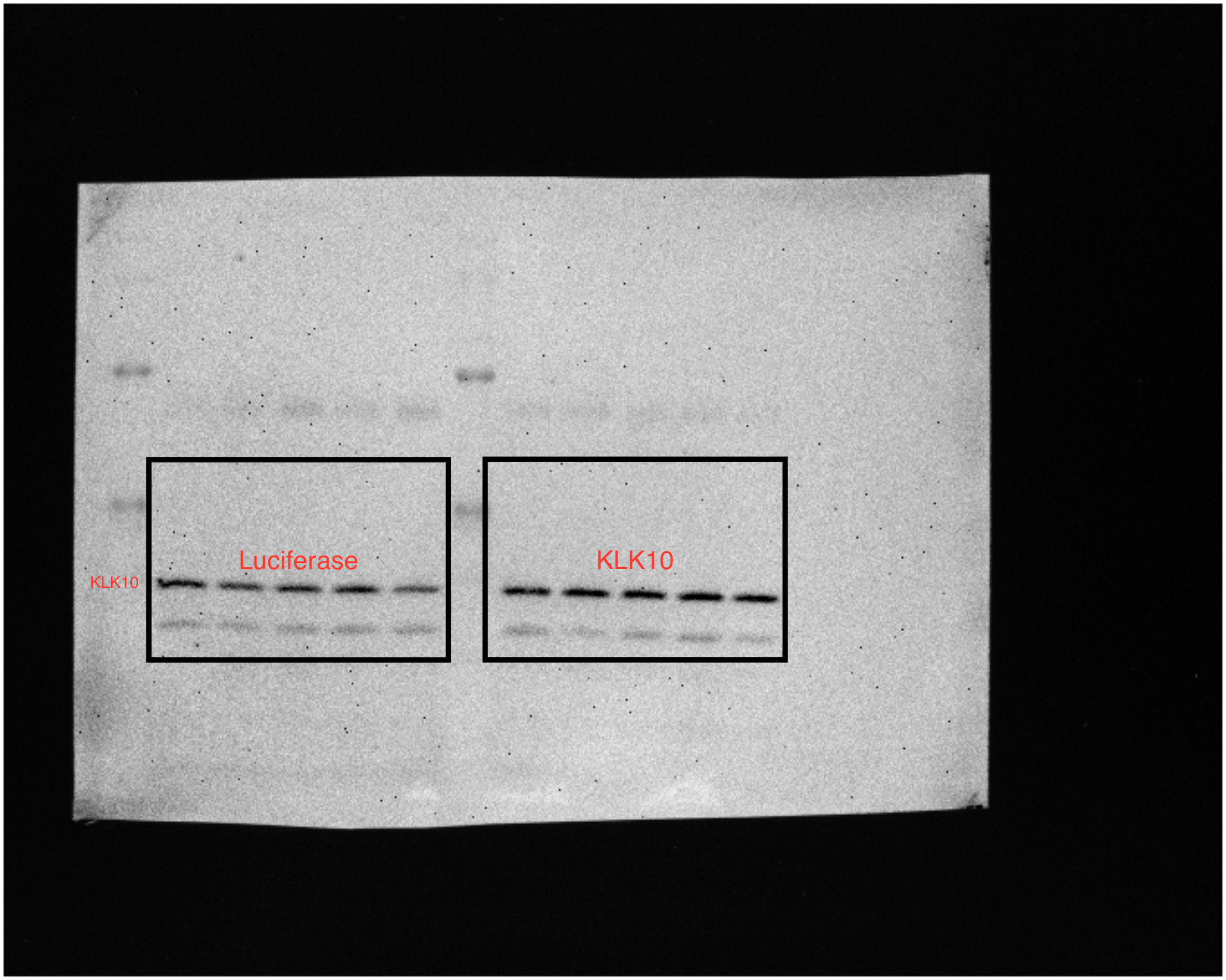

Supplement: Figure 6—source data 1. [file elife-72579-fig6-data1.zip › Figure 6- Source data 1/Figure5h-KLK10_composite.tif]

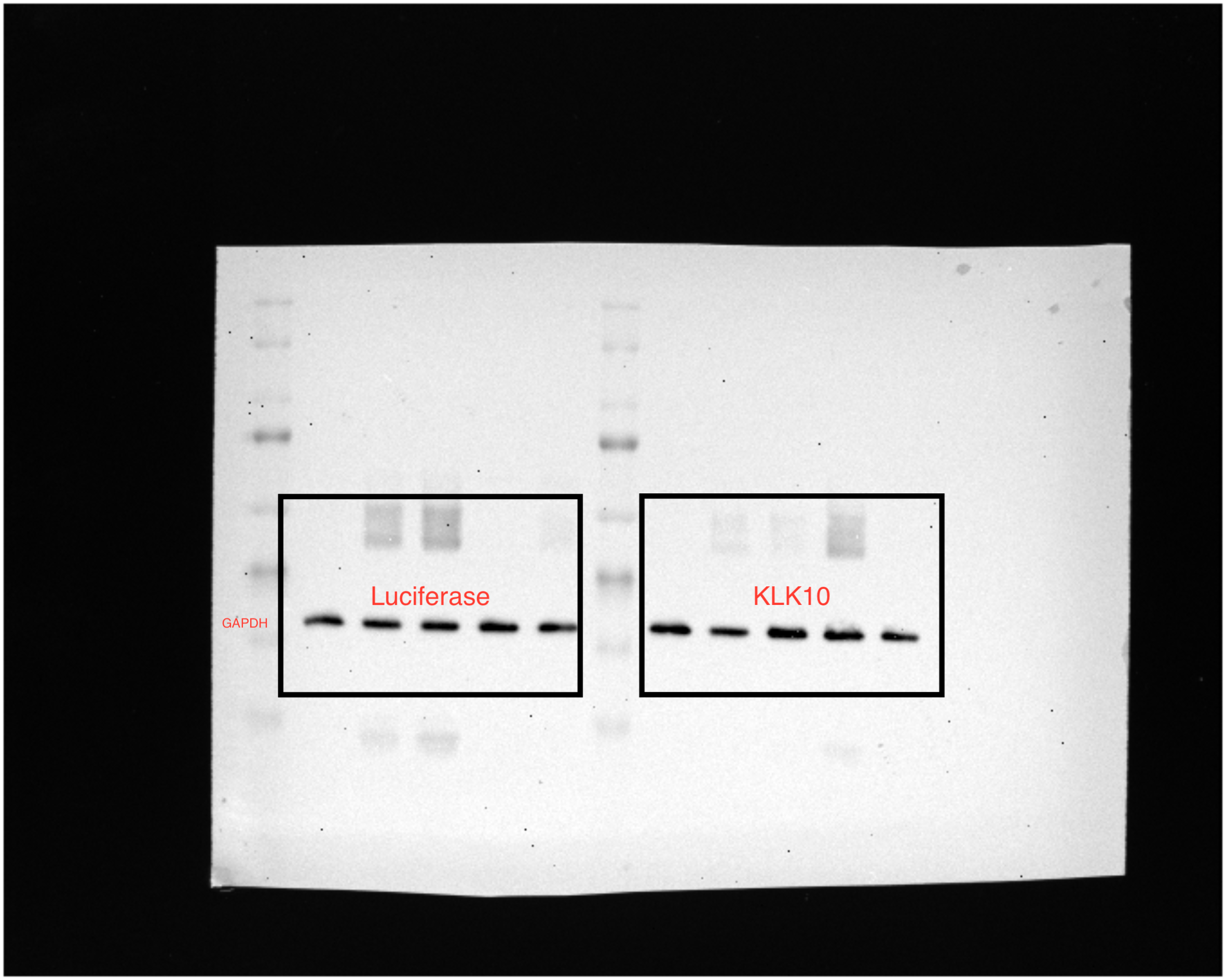

Supplement: Figure 6—source data 1. [file elife-72579-fig6-data1.zip › Figure 6- Source data 1/Figure5hGAPDH_composite.tif]

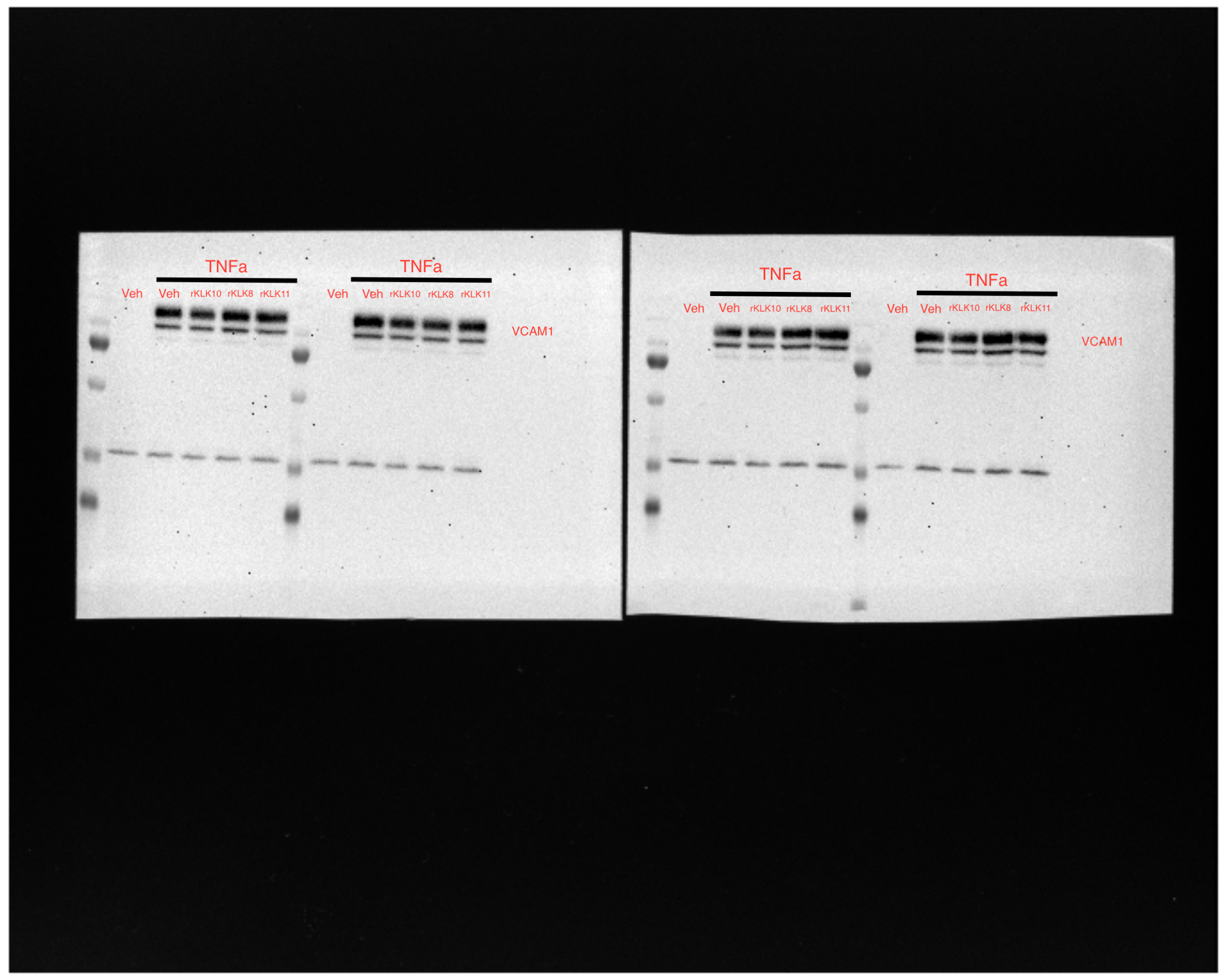

Supplement: Figure 8—figure supplement 2—source data 1. [file elife-72579-fig8-figsupp2-data1.zip › Figure 8- Supplement Figure 2- Source Data 1/Figure 8- Supplement Figure 2- Source Data 1.tif]
